# The promise and pitfalls of community-based monitoring with a focus on Canadian examples

**DOI:** 10.1007/s10661-022-10841-y

**Published:** 2023-03-06

**Authors:** Abdullah Al Mamun, David C. Natcher

**Affiliations:** 1 Department of Agricultural and Resource Economics, 51 Campus Drive, Room 3D08, Saskatoon, SK S7N 5C8 Canada; 2 Department of Biology, University of Saskatchewan, Science Pl, Saskatoon, SK S7N 5C8 Canada

**Keywords:** Community-based, Indigenous, Monitoring, Ecosystem, Resources

## Abstract

Community-based monitoring (CBM) is a widely used form of scientific data collection in which local community members directly participate in ecosystem research and also help the process by sharing traditional ecological knowledge and local understanding of land and resources. This paper presents a review of the challenges and opportunities associated with CBM projects in Canada and internationally. While Canadian cases are the primary focus, international examples are drawn on to provide additional context. Based on our review of 121 documents and publications, we found that CBM helps fill science research gaps by providing access to continuous data sets on the ecosystems being studied. CBM also increases the credibility of the data among users, as the community itself takes part in the environmental monitoring process. CBM supports cross-cultural learning and the co-production of knowledge by using traditional ecological knowledge with science, thereby enabling researchers, scientists and community members to learn from one another. Our review suggests that although there are multiple successes, CBM faces several challenges that constrain its progress including funding shortages, lack of support for local stewardship, and inadequate training for local users in the operation of equipment and data collection methods. Data sharing and rights on the use of data are also constraining to the long-term success of CBM programs.

**Supplementary Information:**

The online version contains supplementary material available at 10.1007/s10661-022-10841-y.

## Introduction

The degradation of natural resources (diminishing water quality and loss of wildlife, fisheries, and overall degradation of biodiversity) have become a key concern at the local and global levels, with implications for development, community economies and livelihoods, Indigenous rights and entitlements, and ecosystem management. These are often the key concerns of sustainable development. However, there are no clear-cut solutions to supporting the sustainable development of natural resources, as multiple interrelated social, political, economic, and ecological factors affect the outcomes of resource governance. Correlating this complexity with the need for sustainable management, solutions to natural resource problems should be sought through engaging diverse techniques, resources, and knowledge systems. In this regard, community-based monitoring (CBM) has come to the fore to address the gaps in ecosystem research and compensate for the shortcomings in science-based monitoring such as short project durations and low numbers of data sets gathered by managers and scientists (Arlinghaus et al., 2017; Conrad, 2006; Castleden, 2015; Eicken et al., 2021; Kanu et al., 2016; Koehler & Koontz, 2008; Lyver et al., 2016; Main, 2011; Mclean, 2014; O’Connor et al., 2005; Reed et al., 2020).

CBM is a term that falls under the “citizen science” approach to resource governance, as local knowledge and community efforts are used to implement the process (Galbraith et al., 2016; Peters et al., 2016). CBM has evolved with the notion of co-management where scientists and managers create working relationships with local communities to address natural resource problems. This approach supports knowledge integration and offers opportunities for the engagement of users with managers for a given resource (Berkes et al., 2007). For example, community-based fisheries management offers data sets and information helpful to dealing with open water fisheries loss in rivers and floodplains (Verbrugge et al., 2017; Thompson, 2006; WWF, 2013).

Community-based monitoring is credited for being a low-cost approach to data gathering and the co-production of knowledge through partnership development (Cornell Lab of Ornithology, 2015; Nyamoga & Ngaga, 2016). It is a process through which government agencies, industries, academics, community groups, and local institutions collaborate to monitor changes in resource systems and respond to local environmental concerns (Kanu et al., 2016; Whitelaw et al., 2003). CBM can be a small-scale research program such as scientific monitoring of water quality of a lake or river section, or can transcend regional and national boundaries such as the snow and ice monitoring programs in the Arctic (Eilken et al., 2021). In addition to supporting targeted scientific research, CBM has contributed to gathering ecosystem information using the traditional knowledge of Indigenous communities to understand ecosystem changes over long time periods. The latest report published by the International Initiative for Impact Assessment indicates that incorporating local participation and accountability often improves developmental outcomes as it stimulates active citizen engagement in service delivery. Moreover, the application of the local understanding of the observed changes enhances community interest in ecosystem monitoring (Waddington et al., 2019).

In addition to supporting scientific data collection, Canadian CBM cases are known to create local jobs and empower communities to carry out projects using their own capacity such as the First Nations Guardians Initiative (ECCC, 2021). Some examples include the Indigenous Guardian Program managed by Munaqusi Community Based Monitoring Project, Inuvik, NWT (https://www.indigenousguardianstoolkit.ca/program-map); Ahousaht Stewardship Guardian Program managed by the Maaqutusiis Hahoulthee Stewardship Society, British Columbia (MHSS, 2021); and the Metis Nation Saskatchewan which established community monitoring systems by training local monitors to investigate areas of concern and data gaps in climate monitoring (Metis Nation Saskatchewan, 2018). The Canadian Federal Government has also used CBM programs to build nation-to-nation relationships (National Institute of Fisheries, 2019; also more detailed information on the Indigenous Guardian programs can be found by reviewing the following webpage: https://www.canada.ca/en/environment-climate-change/services/environmental-funding/indigenous-guardians-pilot/map.html), and acknowledge the wrongs of colonial occupation such as residential schools and loss of traditional rights and governance while addressing the legacy of trauma to Indigenous communities.

The CBM approach has been used in Canada to support ecosystem conservation, as shown by the Canada-wide water quality monitoring program, although limited participation of Indigenous Nations was ensured through such projects (Conrad, 2006; Derworiz, 2016; Kanu et al., 2016; ECCC, 2018; Peters et al., 2016; Pollock & Whitelaw, 2005). However, there are numerous CBM projects carried out in Canada’s northern region with higher participation of the Indigenous communities (Eikecn et al., 2021; Reed et al., 2020). These examples include Parks Canada’s effort to monitor Wood Buffalo National Park in Alberta (Parks Alberta Environment & Parks, 2017; Parks Canada, 2019) and community monitoring of caribou arranged by the Qikiqtaaluk Wildlife Board (ECCC, 2018).

Global CBM programs allow expertise and money to flow between developed and developing countries for projects in various resource sectors. For example, a leading environmental US-based non-governmental organization known as RARE supports the “Fish Forever” program that promotes community-based conservation of natural resources through using an international network (Rare, 2019; https://rare.org/program/fishforever/). RARE takes a behavioral change approach to conservation and works with coastal communities across Brazil, Indonesia, Mesoamerica, Mozambique, and the Philippines. Community-owned carbon monitoring programs in Southeast Asia, the Polynesian Islands, and Mexico are also supported by CBM efforts (IGES, 2014). The CBM approach to water quality monitoring has been used extensively in the USA, with 351 stand-alone or parent programs and 1675 affiliated programs (Green et al., 2016).

The extensive use of CBM both locally and internationally indicates the importance of this process. There is much literature that reports positive outcomes of CBM programs such as knowledge integration, cost-effectiveness, local job creation, mobilization of funds, creation of creditable datasets, and usage across diverse resource systems (wildlife, forestry, fish, waters, etc.). However, there are still challenges that exist at its operational level irrespective of the nation using this approach. According to the scientific communities involved, the CBM approach faces challenges when local data gathered from CBM projects do not comply with scientific data requirements and are incompatible with existing science-based models (Eicken et al., 2021; McCord, 2013). In addition, there are often concerns by scientists that the data collected may not be scientifically reproducible (Fore et al., 2001). Therefore, science is somewhat resistant to the acceptance of the results of CBM projects. Other research has indicated that although CBM enables local community involvement, it does not bring long-term social and economic benefits to the participating communities (Castleden, 2015; Carlson et al., 2017; Eicken et al., 2021; Ortega-Álvarez et al., 2017; South East Queensland Catchment Authority, 2017; Topp-Jorgensen et al., 2004). Insufficient access to equipment, training, and resources are also considered to be limiting factors for effective CBM (Dickinson et al., 2012).

Canadian CBM programs face unique challenges as a result of colonial regimes that revoked land rights from the Indigenous Nations through signed treaties. Programs such as the Indigenous Guardians were launched with the aim of building relationships between the nations. However, specific goals such as long-term support for youth employment and education are yet to be achieved. Such programs lack sufficient opportunities in these areas for the youth of Northern communities who face a high rate of unemployment and limited access to science curriculums. As per Wong et al., (2020), Indigenous youth are further behind their non-Indigenous peers in receiving science education in Canada. Scaling up of CBM outcomes to include such issues is not often considered. There is also the belief among participating communities that colonial powers still govern the CBM process, as the communities must compete with each other for a small number of funds to operate their CBM programs which limits both the scope of ecosystem research and opportunities for Indigenous governance to take part in CBM process.

Indigenous communities are concerned about the utilization of and establishment of rights over the data generated by these projects, as they are not defined in many regions of the world including Canada (https://fnigc.ca/ocap-training/) and the Indigenous members often do not hold the intellectual property rights (AFN, 2010). The approach often favors science but puts Indigenous knowledge systems at a disadvantage (Carlson et al., 2017; Eicken et al., 2021). Considering these aspects, CBM is an untapped opportunity in addressing provisions made under the UNDRIP (UN, 2007) and the Truth and Reconciliation Commission and supporting Indigenous empowerment (Truth and Reconciliation Commission of Canada, 2012).

The above information suggests that CBM has both promises and pitfalls in its application. The diversity of outcomes generated by this approach has motivated our review of CBM programs both globally with an emphasis on Canada to inform the further application of CBM. Given this understanding, we investigated CBM programs covering a range of natural resources including waters, wildlife, fisheries, ecosystem, and climate monitoring available internationally and in Canada. We draw on the lessons, experiences, and outcomes of a wide range of past and present projects covering various ecosystems and natural resources. Our intention was not to present a systematic review or to provide a detailed account of certain projects or any resource system but to develop an understanding of CBM context by exploring the challenges and opportunities of this form of ecosystem monitoring in natural resource sectors.

## Materials and methods

Our review was informed by a synthesis of trends and gaps in CBM cases globally and in Canada. We asked the following questions:

1. What does the literature say about CBM in the context of the global loss of biodiversity including the degradation of water quality, forests, wildlife, and marine resources?
2. What is the progress made so far in implementing CBM internationally and in Canada?
3. What are the challenges that limit the further use of the approach?
4. What recommendations can be made to further community engagement in CBM projects?

To answer these questions, we examined published literature and various online resources, including project-related webpages, to document available CBM examples and related outcomes. A two-pronged desktop review of online materials focused on regional, national, and international CBM projects was conducted between April and July 2017 (the first round of information gathering and review), and May 2021 to January 2022 (the second round that covers new cases and recently published scholarly works). First, we examined international CBM outcomes in both developing and developed nations, for example, water quality projects in the USA (Green et al., 2016), coral reef conservation in New Zealand (Peters et al., 2016), wildlife management projects in Hawaii, USA (Friedlander et al., 2010), and newly evolved carbon monitoring through the REDD + program which focuses on developing nations such as Vietnam and Indonesia (Ferrari et al., 2015). Second, we reviewed documents on Canadian CBM programs, such as water quality studies on lakes and rivers near mining and other extractive resource sites. The review also involved consulting the repository of the Athabasca River Basin managed by the Athabasca River Basin Research Institute (2017), which harbors a collection of published literature on water quality in Canada. This stage of the review helped to identify the breadth and coverage of CBM programs at regional levels. In all cases, we focused on the performance of community-based organizations, the types of projects they support, and the level of community involvement.

To start the survey process and internet-based screening of the CBM cases, a keyword-based search was performed, which included “community-based monitoring” and related words such as “community-based monitoring Alberta,” “community-based monitoring fisheries,” “community-based monitoring waters,” “community-based monitoring Arctic,” “community-based monitoring lakes in Alberta,” and “community-based monitoring forest.” Other Google searches focused on related issues such as community-based monitoring challenges or benefits.

The results obtained were organized using a spreadsheet with the column headings as shown in Table 1:

**Table 1:** Structure of the spreadsheet used to organize CBM programs data

Program	Country	Region	Community	Physical settings	Species/resources	Links	Funding	Project descriptions (notes)

**Fig. 1:**
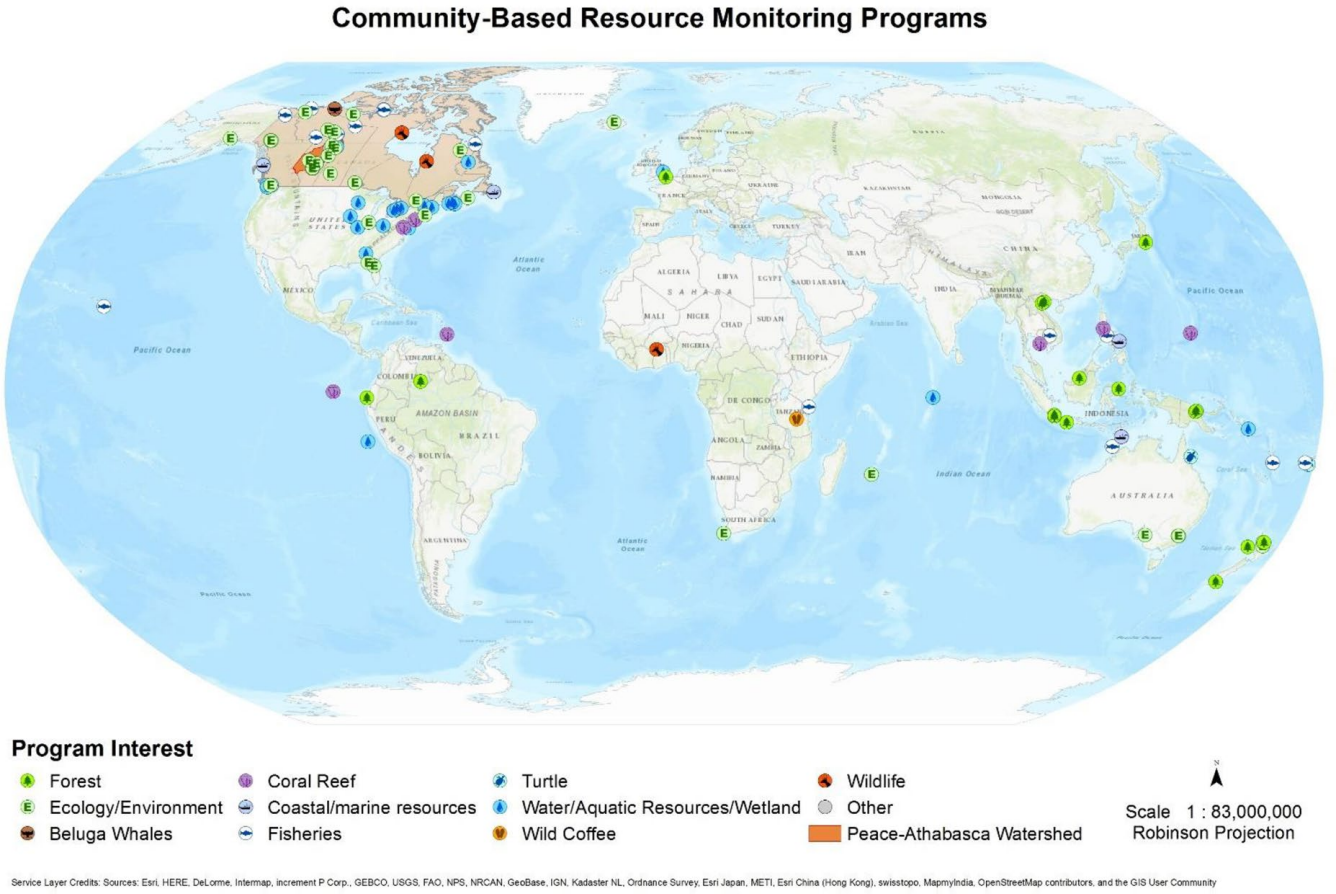
Illustration of CBM contributions across different resources in Canada and at global levels. The rationale behind the projection of diverse resource systems across different geographic regions in the map is to demonstrate the fact that CBM is a popular approach and has a global distribution, can operate across nations with dissimilar governance and economic structures, and can contribute to conservation and management of ecosystem values (forests, wildlife, fish, waters, etc.) that are threatened/degraded by human and natural disturbances such as overuse of resources or climate change impacts on them. However, these are the projects we described in our review to support our interpretation and analysis. We reviewed all the CBM projects and their target resource systems and found that we could broadly categorize them for our use

The summary presented in this report includes project activities, origins, communities, funding information (if available), and updates on the benefits and challenges of CBM projects. While gathering information on CBM cases, we plotted the geolocations of the projects in a separate file. This data was used to create a Geographic Information System (GIS) map showing the distribution and concentration of the cases reviewed (Fig. 1). It must be noted that when similar projects are managed by an organization, only its main location was used irrespective of the actual project locations. This was done to avoid clumsiness in the mapping and to group analogous approaches. For example, in the cases of the Centre for Indigenous Environmental Resources (CIER) Canada, we used only its main location of City or Country (CIER, 2017). Similarly, in the US cases, we considered only the water monitoring projects at the regional level such as the eastern zone of the USA, although several water monitoring efforts exist (Green et al., 2016). We provide a supplementary index based on Google search to acknowledge the contributions of local/Indigenous participants in CBM programs (see Annex-1 of the paper).

Although projects are all unique in their functions and produce diverse outcomes, for the purpose of this review and to facilitate visualization, we broadly group them as forests, coral reefs, turtles, wildlife, ecology, coastal and marine resources, beluga whales, fisheries, and wild coffee production. However, we note that this classification is arbitrary and less scientifically sound.

Although a vast amount of data was collected, interpretations of the significance of CBM were made based on reflections included in scholarly articles and project websites. We present the review outcomes of 121 documents at the Canadian and international levels, including published papers and data from related websites that refer to CBM. The selection of documents used in this review was based on criteria such as the provision of clear information on the project outcomes in social (levels of local participation) and ecological (conservation success) terms, inclusion and identification of issues and challenges of CBM, and suggestions for furthering the projects. Therefore, we caution that the outcomes listed in this paper refer only to those presented by academic researchers in their articles and not those of the communities engaged in such research. Further research should examine community perspectives on CBM projects for a more comprehensive understanding of project outcomes.

## Results and discussion

The results are presented in three subsections. The first subsection (State of CBM approach) includes an update on the geographic distribution, resource coverage types, and overall activities of CBM projects and project-level outcomes as indicated by researchers. The second subsection outlines the benefits and opportunities afforded by of the CBM approach as discussed in the examined materials. The third subsection describes the challenges faced in CBM implementation. All the sections also contain necessary information including a map and several tables as required to present the related materials formatively.

### State of CBM approach: overall

Both Canadian and international cases are reviewed to accomplish the objectives of the paper. The international CBM projects we reviewed include wildlife (African regions), water and wetlands (USA), forests and ecosystems (Africa, Brazilian Amazon, USA, Southeast Asia, Australia), and marine resources such as coral reefs and coastal fisheries (Australia, Fiji, and Peru). Our research shows that Canadian and international CBM programs cover a range of projects and resource systems including wildlife, fisheries, and ecosystem values (Fig. 1). The projects we reviewed are included in the visual presentation depicting their geographic locations in Fig. 1. We found that international and local CBM projects were performed to fill information gaps by adding increased levels of data to support projects involving natural resources, species-at-risk tracking, and protected area conservation (Davidson, 2016; Green et al., 2016; Conrad & Hilchey, 2011). CBM projects also increase community knowledge, as community members learn about the ecosystem they utilize (Reed et al., 2020), and are able to actively participate in project activities such as equipment operation and data collection (Kanu et al., 2016; WCS (Wildlife Conservation Society) Canada, 2018).

In light of its multiple benefits, all developed and developing countries have endorsed CBM for managing a diverse set of resources. Our study shows that diverse CBM programs exist in various parts of the world, with a higher concentration in developed nations (Conrad, 2006; Conrad & Hilchey, 2011; Kanu et al., 2016; Alberta Lake Management Society, 2021). Developed countries like the USA (Green et al., 2016) and Australia (Edwards, 2015) have embraced CBM extensively. The USA has a public forum to support the community-based monitoring of waters (Florida Lake Watch, 2013). Voluntary countrywide water monitoring to address quality concerns from industrial and agricultural pollution has been taking place in the USA for decades. Green et al. (2016) recorded more than 300 CBM cases across the USA with a focus on water quality monitoring.

Australia is another pioneer in utilizing CBM, with nationwide Australian Rangers programs that recruit Indigenous members to conduct ecosystem research (Australian Government, 2017; Traill, 2017; Peters et al., 2016). As of 2018, this program supports ranger groups across Australia and funds 831 full-time jobs. Ranger groups monitor dugong and sea turtle populations, and support traditional wildfire reduction activities (Leach, 2018). Danielsen et al. (2014) identified 170 community-based monitoring programs (fisheries, forestry, sea ice, climate, etc.) in the Arctic from the peer-reviewed literature and from searching the internet. In addition, developing countries use CBM as a low-cost and community-driven approach run mostly through donor funding. For example, Bangladesh, Burkina Faso, Cambodia, Pakistan, the Philippines, Nepal, Vietnam, Senegal, Sri Lanka, Benin, Ghana, India, Laos, and Indonesia are operating various fisheries and natural resource monitoring projects including forestry (Garcia & Lescuyer, 2008; IGES, 2014; Ryes, 2005). The Philippines has the Fish Forever Program, which also undertakes community coral reef management (Rare, 2019). Fiji’s coral reef monitoring project supports community-based turtle conservation.

Through these programs, communities gain the knowledge required to scientifically assess measures of ecosystem health such as carbon content and can access global carbon funds (IGES, 2014). All of these projects support conservation through low-cost data gathering and management. For example, Fiji’s voluntary conservation program has benefited communities, as it promotes self-monitoring. It operates with a small budget of US$4000 per year, which covers data analysis, training for monitors, and the synthesis and interpretation of the results (Breckwoldt & Seidel, 2012).

Similarly, Canada has a number of CBM and community engagement programs. These include Guardian programs that cover local harvest monitoring, Inuit biodiversity monitoring, and long-term species monitoring studies (ECCC, 2021). Indeed, waters are a common area of CBM intervention, with around 180 ongoing related programs in Canada (Carlson et al., 2017; Conrad & Hilchey, 2011). The Canadian government also supported 14 CBM initiatives in the Arctic, where local monitors work with university and state department experts to gather data. Boats and tool kits are made available to participating communities (Government of Canada, 2017).

These programs were supported to build relationships between Indigenous and government agencies. More significantly, the Canadian government has arranged national programs to fund local CBM initiatives, such as Guardian Watchmen programs that are supported through fiscal budgets (Coastal Guardian Watchmen, 2017). Similarly, conservation programs led mainly by the Indigenous leadership and the Guardians work for the programs called “eyes and ears” on traditional territories (https://www.ilinationhood.ca/guardians) are operated to create models for Indigenous-led monitoring activities with the guidance of Elders. The Guardians are trained Indigenous experts to manage protected areas, restore animals and plants, test water quality, and monitor development. Its partners are working to create a network to coordinate their activities and information/knowledge sharing among Indigenous-led CBMs. Guardian programs include land-based learning, hands-on case studies, culture, arts, and community dialogue (Baker, 2021; Arctic Borderlands, 2014; Reed et al., 2020; https://www.banffcentre.ca/programs/introductory-wise-practices-indigenous-leadership-online/20220322). Indigenous Guardian Programs are considered vital to achieving conservation goals as they directly involve remote Indigenous communities, integrate traditional knowledge to fill the gaps in management decisions, and improve understanding of ecosystem processes.

Regional and provincial cases of CBM are also evident in Canada indicating community participation in programs that have environmental concerns. The present information suggests that community participants were key members of CBM teams, collecting data to perform research in programs such as the Alberta Oil Sands Monitoring Panel 2016 (AEMERA, 2014; Hopke et al., 2015). Private organizations like Canadian North Environmental Services (CanNorth) have specific programs to understand mining impacts on the ecosystem and human health such as Eastern Athabasca Regional Monitoring Program (CanNorth, 2017). In addition, CIER has greatly supported CBM operations in the North by engaging in various lake monitoring programs that involve Aboriginal communities and creating documents such as toolkits to facilitate the CBM process (CIER, 2012, 2017). At the regional context, the Keepers of the Athabasca (2011) organized community-based monitoring along the Athabasca River, Peace-Athabasca Delta, and Slave River Delta to address concerns about unhealthy fish that were caught in the area. These fears were related to the upstream development of oil sands and hydroelectric facilities and were complicated by climate change.

In addition, a shift in the collection and monitoring of data has occurred in Alberta, particularly when provincial records on water quality and fisheries were challenged by other data sets, such as those used by private organizations like LakeWatch Alberta Programs (Alberta Lake Management Society, 2021). Gérin-Lajoie et al. (2018) note an increasing interest in community-based environmental monitoring (CBEM) in Northern Canada in response to the rising impact of resource exploitation and climate change, and due to the increased recognition of Indigenous knowledge. For example, Alberta now has several CBM programs, including the Regional Aquatics Monitoring Program (Main, 2011; RAMP, 2015) and Peace-Athabasca Delta Ecological Monitoring (PADEMP, 2021). The RAMP covers the Athabasca River and its tributaries, the Athabasca River Delta and regionally important lakes and wetlands. It monitors climate and hydrology, precipitation rates, air temperature, snowpack measurements, and water quality. It focuses on determining the potential exposure of living aquatic organisms to various chemicals and water conditions. Its other programs include detecting benthic invertebrate communities, sediment quality, and fish populations.

Our observations suggest that most of these projects in Canada operate as partnerships. For instance, the University of Saskatchewan promotes programs in Alberta and the Northwest Territories such as Slave Watershed Environmental Effects Program (SWEEP) (http://sweep.insighthosting.com/about.aspx). An Aboriginal organization called CanNorth has established a multiyear East Athabasca Environmental Monitoring Program (EAEMP) to address ecosystem and human health issues related to uranium mines in the Athabasca region (CanNorth, 2017). This region has limited road accessibility, making it difficult to monitor abandoned uranium mines for untreated ore, which is a source of radon contamination. Government projects are also wide ranging and include mercury testing initiatives in Canada’s Northern lakes (Environment and Climate Change Canada, 2018). The programs operated by CIER and CanNorth also serve to bridge local communities with state-driven programs in Canada. This volume of CBM projects attests to the significance of the approach in international and Canadian contexts.

Other aspects of CBM involve the applications of diverse techniques and approaches which have resulted in multiple activities while implementing CBM cases. In Table 2, we provide a summary of the general activities of Canadian and international CBM programs and their key outcomes including their activities and performance. We also provide information on key local and global cases that cover multiple regions and resource systems. In this regard, we included related information to characterize the programs such as outlining their key activities, roles of members to operationalize CBMs, and overall outcomes.

**Table 2:** Summary of overall activities and outcomes of selected Canadian and global cases

Project names and areas	Key activities	Outcomes
*Canadian cases*		
Wildlife Conservation Society (Raygorodetsky & Chetkiewicz, 2017)	Brought people together to share perspectives of different ways of knowing about the land and aimed at better understand the changes on ecological and social systems in northern Ontario	Demonstrated the value of engaging local communities summarizing various project outcomes in ecosystem monitoring assisted by modern drawing tools (https://www.upnorthonclimate.ca/community-based-monitoring)
Slave Watershed Environmental Effects Program (SWEEP, 2018)	Monitors fish and wildlife health in Athabasca region to detect if fish are safe to eat, if the water is drinkable, and if the ecosystem is healthy	Members helped co-create TEK and Western science indicators of environmental change using a “two-eyed seeing” approach (http://www.integrativescience.ca/Principles/TwoEyedSeeing/)
Regional Aquatics Monitoring Program (RAMP, 2015); AEMERA (2014)	Monitors the aquatic environment in the oil sands region of Alberta for potential concerns	Communities monitored fish populations (abundance, growth, and tissue quality) as a biological indicator of ecosystem health and integrity
Arctic Borderlands (Indigenous driven, 2017; 2014)	Works in Alaska, Yukon, and the Northwest Territories to address ecosystem management and environmental health issues	Used local ecological knowledge to monitor caribou herds in northwest Arctic Canada. Member organizations collect ecological data to empower communities (https://www.arcticborderlands.org/)
Andrachuk and Armitage (2015); Northwest Territories (NWT) Water Stewardship	Act as advocates, support traditional knowledge collection and application, and design research and monitoring programs	Measures water quality, water quantity, groundwater, and biological components in the NWT, including cumulative impact assessments (https://www.nwtwaterstewardship.ca/)
Indigenous Wisdom Advisory Panel Alberta	Uses traditional knowledge to interpret changes in the environment around Aboriginal communities across Alberta	Promotes a consensus-based process for giving collective advice and recognizing both oral and written communication. Works to achieve commitment to the United Nations Declaration on the Rights of Indigenous Peoples (https://www.alberta.ca/indigenous-wisdom-advisory-panel.aspx)
CanNorth Community Programs	Indigenous community-owned approach with programs that engage and consult with communities and First Nations nationally and internationally to address environmental issues	Focuses on consumption and safety of traditional foods in mining areas. Runs five programs in First Nations communities in Alberta and Northern Saskatchewan (https://cannorth.com/community-programs)
Arlinghaus et al. (2017); Centre for Indigenous Environmental Resources (CIER, 2017)	Develop and implement sustainable solutions to proactively address environmental issues affecting First Nations lands. Ensure security, availability, and quality of our freshwater and the ecosystems, people, and communities that depend on it	Worked with over 350 environment-focused projects with over 300 First Nations across Canada (http://www.yourcier.org/; http://www.yourcier.org/uploads/2/5/6/1/25611440/icbcm_symposium_cbm_slides.pdf)
Indigenous Guardians Programs	The largest forum of Indigenous communities, with 43 member nations across the Canadian North	Members are treated as the “eyes on the ground” in Indigenous territories to monitor ecological health, maintain cultural sites, and protect sensitive areas and species (https://www.indigenousguardianstoolkit.ca/program-map)
Peace-Athabasca Delta Ecological Monitoring (PADEMP), Alberta and Yukon	Local members track changes to the water and land in the traditional areas of the Mikisew Cree and Athabasca Chipewyan First Nations	Resources, technicians, and labor are shared among communities when needed (PADEMP, 2021 https://www.3ne.ca/wp-content/uploads/2020/06/Delta-Fact-Sheet-General-2020-06-05-e.pdf)
Indigenous community-based climate monitoring	Canadian National Programs available in all provinces/territories in Canada with a focus on Canadian North	Programs have focused on empowering Indigenous communities to take part in climate change mitigation by measuring indicators such as water temperatures, oxygen, pH, and conductivity in fish-bearing lakes by Chipewyan Prairie Dene First Nation. The Métis Nation of Alberta Community-Based Climate Monitoring Initiative identified priority climate indicators and traditional land use areas vulnerable to climate change (https://www.rcaanc-cirnac.gc.ca/eng/1594046483192/1594740453550)
Coastal resource Observation network	Coastal Guardian Watchmen, British Columbia	An early Guardian program (2005) and it operates with the assistance from several coastal nations and plays a critical role in all aspects of stewardship for Coastal First Nations including conservation of natural resources (http://coastalguardianwatchmen.ca/guardian-watchmen-programs-overview)
*Global cases*		
Indigenous Ranger Programs: Australia	Support Indigenous people to combine traditional knowledge with conservation training to protect and manage their land, sea, and culture	Key activities include bushfire mitigation, protection of threatened species, and biosecurity compliance (https://www.niaa.gov.au/indigenous-affairs/environment/indigenous-ranger-programs)
REDD + projects: Multi-country	Focus on developing nations addressing forest resources degradation	Key activities include forest restoration and sustainable management of forests through providing training and technological supports. Purchased lands have high conservation values (https://www.worldlandtrust.org/what-we-do/ and https://www.fieldmuseum.org/blog/thoughts-reducing-emissions-deforestation-and-forest-degradation-redd)
Community-Based Monitoring in Arctic (Arctic)	Arctic Council supported monitoring programs	Focuses on gathering climate indicators considering the changes in flora and fauna in Arctic Regions using Indigenous knowledge. Local community can use their knowledge to solve their local problems such as changes in caribou hunting grounds (https://arctic-council.org/news/community-based-monitoring/)
Local Environmental Network, Alaska	US Arctic programs such as Bering Sea Environmental Monitoring Network for Adaptation and Security	Combines local communities and uses observer blogs to support two-way communications to record changes in Arctic (https://arctic.noaa.gov/Report-Card/Report-Card-2015/ArtMID/5037/ArticleID/223/Community-Observing-Arctic)
Community-based fisheries monitoring project Bangladesh	A UK–Bangladesh joint CBM initiative to support wetland management	Promotes empowering marginalized to assert their rights over traditional fishing grounds and offers conservation training (http://pubs.iclarm.net/resource_centre/WF_778.pdf)
Participatory Community Based Monitoring Benin	World Bank knowledge-sharing programs concerning coastal and marine biodiversity	Aims to maintain the biological diversity and ecological functions of coastal wetlands and other ecosystems in the coastal zone, while supporting the livelihood and economic opportunities of the communities living in these areas (https://documents.worldbank.org/en/publication/documents-reports/documentdetail/555461468005483145/benin-community-based-coastal-and-marine-biodiversity-management-project)
Community-Based Forest Ecosystems Management in Tanzania	USAID supported forestry program	Focus on providing ownership of forest and wild to local users: https://ghanalinks.org/documents/20181/0/Lessons+on+Best+Practices+of+Community-based+Ecosystems+Management+in+other+Parts+of+Africa/13d06546-0d38-4945-a87a-4dc0f533a633

### Specific examples of positive outcomes as benefits of CBM

Canadian and international CBM cases with diverse activities produce multiple outcomes. Our review of the literature found that while CBM offers numerous benefits in both international and local Canadian projects, there are associated challenges and obstacles that affect their outcome. In the following two sections, we describe this in terms of positive outcomes as benefits and negative outcomes as challenges. We present the outcomes of international CBM projects first, followed by Canadian CBM projects.

### Examples of CBM benefits: global cases

A review of the global cases shown in Table 3 below suggests that CBM has various positive social and ecological outcomes. These include local participants receiving environmental training that increases literacy, greater community involvement in decision-making, helping communities to manage their lands and resources, and protection of wildlife (IGES, 2014). CBM cases at the international level indicate that CBM is beneficial to resource users, as it helps them participate directly in field data collection to support the conservation and management of natural resources (Fernandez-Gimenez et al., 2008; Gérin-Lajoie et al., 2018; Weston & Conrad, 2015; Van Rijsoort & Jinfeng, 2005). CBM enhances the accountability and transparency of research projects through partnership, such that local participants are able to see the changes happening in their landscapes (Waddington et al., 2019). It also fosters community pride and enhances social values and efficacy as is evidenced by IGES programs across many parts of the world. IGES projects have helped stop illegal logging in many islands in Indonesia and the Philippines (IGES, 2014). Sometimes, CBM-related services are voluntary for enhancing environmental stewardship, while interactions between participants can engender a stronger sense of community and shared purpose (Lawrence, 2006). In 2015, a Fiji community has raised US$2000 to support training for local members to conserve coral reefs (Coral Reef Alliance, 2016), and the coral reef initiative in Fiji is known to establish self-governance (Tang, 2012).

**Table 3:** Summary of key project outcomes as strengths and advantages on a global level

References	Region/CBM		Outcomes and strengths
*Global*			
Commodore et al., 2017; Danielsen et al., 2009		Global: multiple resources	Bottom-up approach with observing requirements tied to management outcomes that community members or institutions both appreciate
Danielsen et al., 2014; Singh et al., 2014		Global: multiple resources	CBM supports better social and economic outcomes. It is a cost-saving approach, as the investment in volunteer time to collect data is much less than agency administration costs. The volunteers live locally and often support the CBM. This inculcates social values like knowledge sharing
Singh et al., 2014; Trumbull et al., 2000		Global: multiple cases	The social outcomes of volunteer participation in monitoring include improved scientific and environmental literacy and greater community involvement in decision-making
Forest Peoples Program Guyana, 2012		District Toshao Council: Guyana forests	Communities have control of their lands through building their own capacity. The community plan details hundreds of local wildlife sites for protection
Lawrence, 2006		Global: biodiversity	Volunteers’ field-based activities can function as a catalyst for enhancing stewardship and support land-based learning, while interactions among participants can engender a stronger sense of community and shared purpose
Leach, 2018		Australia: feral cat, dugong, coral reef, and wildfires	Local jobs, community pride, training and education
Coral Reef Alliance, 2016; Tang, 2012		Fiji: coral reefs	Low-cost monitoring, community funding to support training and education, self-governance
Rare, 2019		Global: Fish Forever	Promote partnership through linking scientists and communities remotely. For example, the University of Santa Barbara has a project in the Philippines for coral reef management through community participation. Our observations suggest that most of these projects in Canada operate as partnerships
Pocock et al., 2014; IGES, 2014		Global south	Support the use of technologies such as drones and cellphones, and training of local people to monitor forest biomasses which brings social pride and boosts empowerment
Evangelista et al., 2018; Young, 2018		African Region	Facilitate multi-species monitoring and modeling focusing on goats and wild asses in Somaliland that would not be possible without local supports and technology adaptation (Traditional knowledge and remote sensing)
Eicken et al., 2021		Global	CBMs act as a cost-saving approach to monitor areas that have less accessibility and where logistics are expensive
Fisheries: Thompson, 2006; Eicken et al., 2021		Global south	Knowledge transfer and fund mobilizations from developed to developing countries to support natural resource programs such as community-based fisheries management programs in Bangladesh
ECCC, 2021		Canadian: Guardian Programs	Allows cultural values help frame community conservation objectives. Nation-nation relationship–building approach (Canadian Government and Indigenous Communities) and Indigenous community empowerment. Focus on relating Indigenous relationship with the land and resources using own powers and knowledge
CIER (2017) and CanNorth (2017)		Canadian NGOs environmental programs	Create opportunities for nongovernmental institutions to work as bridging organizations through CBM programs focusing on Canadian North and Indigenous Nations (links community with government-supported ecosystem projects)
SWEEP Program (2018)		Canada (Alberta)	Links academic institution with Indigenous communities to support local data gathering through technical support
Asselin & Basile, 2012		Canada (Nunavut)	Joint investigations can resolve conflicts between data providers and users by developing data-sharing agreements such as in the Inuit context

Adapted from Conrad and Hilchey (2011) and our own interpretations.

Through this approach, state departments can curtail the costs of monitoring activities, as the services provided the community are often voluntary or involve fewer expenditures (Eicken et al., 2021). In some cases where government funding is available, the cost of operating CBM is far less compared to provincial monitoring, due to reduced overhead costs. This is because the community members collect data where they live, while managers need to travel long distances to reach the fields and set up monitoring stations, and then make periodic visits to collect the data. In some remote areas such as the Canadian Arctic with harsh winter conditions, maintaining monitoring activities becomes more complicated for southern scientists (Johnson et al., 2015, 2016).

### Examples of CBM benefits: Canadian cases

Like the global examples, the Canadian cases (Table 3) also suggest that CBM helps fill research gaps by collecting environmental data on ecosystem health and by providing information from traditional knowledge (Carlson et al., 2017; AEMERA, 2014; Parlee et al., 1998). Most significantly, if the communities do not participate in the research, their traditional knowledge may not be available to science. For example, Inuit in Arctic Canada has become a vital source of information on past mass mortality of avian fauna due to cholera outbreaks (Henri et al., 2018). This situation may compel legislators to make decisions based on limited data (Peters et al., 2016). CBM consistently gathers more data than science-based monitoring and can cover larger, often inaccessible areas like Northern Canada, where there are few roads (Conrad, 2006; Kanu et al., 2016). The current information suggests that science-based data collection is often intermittent and can only address a limited number of habitats and ecosystem properties, while coverage from CBM is generally more extensive (Casey et al., 2016). For example, the government of Alberta has been monitoring the health of fish and aquatic ecosystems since the 1940s. However, these efforts were not exhaustive in terms of the coverage of bodies of water and the severity of the problem (Casey, 2011; Zurawell & LeClair, 2011). Engaging local members in data collection has improved the process (Alberta Lake Management Society, 2021).

Authors of Canadian CBM have identified other advantages associated with CBM. For example, it offers effective communication among resource users and scientists and helps develop the comprehensive modeling of landscape features (Buckland-Nicks, 2015; Parlee & Nation, 1998; Raygorodetsky & Chetkiewicz, 2017). CBM, which promotes co-learning and engaging in common activities, also improved cross-cultural learning for members of the research team who were unfamiliar with the lifestyles of Indigenous communities including Inuit of Arctic Canada, and has been seen as a process of self-governance (Brunet et al., 2014; Natcher & Brunet, 2020). According to Asselin and Basile (2012), to foster the success of the project, investigators can resolve conflicts by developing a data-sharing agreement that creates an understanding of research ethics, such as within the Inuit context. Finally, interactions among participants can engender a stronger sense of community-driven activities through volunteering and help fulfill shared purposes such as Canadian Guardian programs driven by First Nations (Great Bear Initiative, 2017). In some remote areas with harsh winter conditions such as the Canadian Arctic, maintaining monitoring activities becomes more complicated for visiting scientists (Johnson et al., 2015, 2016), thus making CBM an effective alternative. These programs are community driven and use their own ability including traditional ecological knowledge to monitor ecosystem health.

### Specific examples of challenges as limitations of CBM

While CBM has been shown to generate multiple positive outcomes in ecosystem research, this does not mean that it is without its challenges. We share our findings from both global and local projects below.

#### Examples of CBM challenges: global cases

Projects carried out at the international level face challenges in the form of funding, training, and data rights. With respect to Australian cases, a dearth of funding and trained staff, and an unsupportive legal and political environment limit the scope of the process (Peters et al., 2016; South East Queensland Catchment Authority, 2017; Van Hunen et al., 2016). Funding issues are highlighted as a key challenge, and it is evident from the review that when funding ceases, monitoring activities stop (personal experience from community-based fisheries co-management programs in Bangladesh, and also see Thompson, 2006). There are also doubts about the quality of the data collected by local monitors due to inadequate training (Danielsen et al., 2016). The core idea that the community controls data gathering and management decisions are not implemented, such as in the case of forest resource right sharing in New Zealand where CBM was not a success (Storey & Wright-Stow, 2017).

#### Examples of CBM challenges: Canadian cases

Studies done in Canada have indicated project constraints associated with funding in moose monitoring projects (Singh et al., 2014). Unpredictable funding, inconsistent monitoring protocols, insufficient knowledge of local monitors in operating monitoring equipment, and difficulty in translating diverse and regionally specific data into coherent recommendations for decision-makers are also identified as challenges in Canadian CBM cases (Carlson et al., 2017). Moreover, Indigenous communities in Canada have concerns about ethical use of data and data ownership, given that the traditionally held knowledge that is used to gather these data cannot be separated from its holders (AFN, 2010). Indigenous communities of Northern Saskatchewan are in fear of losing their data due to the lack of proper measures/protocols that protect them from appropriation. Given this constraint, many Indigenous nations across Northern Saskatchewan is developing their own protocols with the assistance of Prince Albert Grand Council, Saskatchewan (personal communication with Robin McLeod, Stanley Mission, Saskatchewan with Cree Indigenous heritage 2022).

In addition, there is an overall failure to define critical aspects of CBM projects, such as access to data collected jointly or individually, the frequency of monitoring, and the establishment of specific measures to involve the participating community in the research process. There is major concern about the ethical use of the data generated in both international and local cases. For example, although monitoring protocols have been developed for some projects, little is known about how effectively they facilitate the collection of data, data archiving and ownership rights that support the groups’ restoration objectives (Pollack & Whitelaw, 2005). However, there are a few exceptions, such as the Prince Albert Grand Council (https://www.pagc.sk.ca/), a Northern Saskatchewan Tribal Council that has been working on protecting its own data by creating Indigenous knowledge/information sharing protocol and creating their secured webpage to maintain/preserve their land-based information as the source of traditional ecological knowledge (personal experience; McLeod, 2021). Researchers working on evaluating international cases have raised doubts about the actual outcomes of CBM projects with respect to the quality of the data and have suggested training as a means to overcome its potential lack of authenticity (Conrad & Hilchey, 2011; Dickinson et al., 2012).

While evaluating Canadian examples and considering Indigenous responses to the CBM process, we found that research objectives are often poorly communicated to participating communities. With few exceptions, these communities are uninformed about the intent and ultimate benefits, if any, of the projects (personal observation while working with First Nations across Northern Saskatchewan). To avoid those unintended outcomes, communities under the Prince Albert Grand Council (an Indigenous organization representing Cree, Dene, and Dakota communities in northern Saskatchewan) have supported the development of an Indigenous knowledge protocol that advocates local community involvement in all stages of proposal development and project implementation, as well as appropriate remunerations for their Elders’ contributions. They believe that this is the proper way to acknowledge their knowledge systems and traditional rights and entitlement over lands, and should be clearly stated in the submitted proposal. Such protocols would introduce a new method of CBM that makes communities central to research programs and avoids a top-down approach to local engagement and gathering of traditional ecological knowledge (Mamun et al., 2021). Table 4 further summarizes the key challenges identified in both international and Canadian cases through some recent reviews of CBM projects and by our global investigation.

**Table 4:** Summary of the project outcomes as challenges (global and Canadian cases)

References	Region/CBM project	Types of challenges identified
*Global cases*		
Eicken et al., 2021	Global	Often a science controlled approach as academia and government agencies determine target variables and guide implementation of the monitoring network, referencing broad societal benefits without considering local interests. This suffers from the potential disconnect between overall societal benefits and Indigenous interests that contradicts with treaty systems especially in Canadian and Australian contexts
Conrad & Daoust, 2008	Global	Limited funds and competing needs as are the cases with donor-driven projects make it difficult for developing countries to establish long-term monitoring programs
Eicken et al., 2021	Global	An exogenous approach which lacks local innovation as the monitoring framework is informed by global frameworks (such as the Convention on Biodiversity) and often does not fit local requirements. Communities lack the capacity to adapt to regional and global framework
Danielsen et al., 2016	Arctic: fisheries, forestry, herding, hunting, sea ice, caribou, etc	Scientists may ignore locally collected data as subjective and anecdotal despite the growing body of literature that demonstrates that where Indigenous and local knowledge has been systematically gathered, the data collected by community members are comparable to those arising from professional scientists
McCord, 2013	Philippines: coral reefs	The objectives of CBM are often unclear to the community. People ask why they need to participate, as the utility of the data was never properly explained to them
Fore et al., 2001	Global: waters	Doubt exists about the quality of the data that volunteers collect. Data-gathering training for volunteers should be implemented, such as for benthic macroinvertebrate studies
Ortega-Álvarez et al., 2017; Topp-Jorgensen et al., 2004	India and Cameroon: forests	CBM activities are financed by donor-supported projects. When funding ceases, the monitoring stops. The level of access to data collected jointly or individually, the frequency of monitoring, and community needs analysis are not defined
Storey & Wright-Stow, 2017; IGES, 2014	New Zealand: macroinvertebrate monitoring	Governments are reluctant to hand over rich forest resources to communities. It is challenging to reorient forestry away from looking only at trees toward looking at the rights and well-being of the millions of people living in and around forests
Peters et al., 2016; Van Hunen et al., 2016	New Zealand: waters	Interest from the government may decrease with the rising level of data, and freshwater management agencies have made little or no use of it
South East Queensland Catchment Authority, 2017	Australia: waters	The state department may run out of funds due to various external impacts and considerations. Sometimes a lack of funding may make it impossible for the government to maintain the community water monitoring program and a reduction in the frequency of monitoring is likely
Dickinson et al., 2012	Global: insufficient knowledge of local monitors to operate equipment	With the rise of CBM, tool kits and monitoring protocols have been developed, but little is known about how widely these tool kits are used and how effectively they facilitate the collection of data that support groups’ restoration objectives
Costa et al., 2018	Forests (Brazilian Amazon)	CBMs often lack data sharing and access agreements
*Canadian cases*		
Higgins (2016)	Fisheries (Indigenous Guardian program)	Government review indicates that obtaining/maintaining the funding required given inflationary impacts. Lack of trust in some areas means First Nations are reluctant to fully engage. This is likely due to distrust that exists among the government and Indigenous communities
Keats, 2020	Wildlife	Mobilizing knowledge from Indigenous research participants and resource co-management decisions are fraught with issues of knowledge authority and epistemological differences, issues of reductionist representation of Indigenous knowledge, and interdisciplinary tension
ITK (2018)	Canada (Nunavut)	Failure to create regionally appropriate ethics protocols and research agreements to support CBM programs
Kanu et al., 2016	Waters	Challenges include a lack of appropriate monitoring protocols, cultural differences between Indigenous participants and scientists, differences in understanding and interpreting different forms of knowledge, the ability to translate this knowledge into decisions, lack of local motivations, inconsistent data format and accessibility
Carlson et al., 2017	Waters	Inadequate or unpredictable funding, and difficulty in translating diverse and regionally specific data to coherent recommendations for decision-makers
Singh et al., 2014	Canada: moose monitoring	CBM implementation is often constrained by a lack of finances and community motivation, inadequately trained staff, and unsupportive legal and political environments

## Conclusion

Considering the processes applied to operationalize CBM across diverse resource systems (Fig. 1, Table 2), advantages and disadvantages of the CBM projects as shown in Tables 3 and 4, and the information presented throughout the paper based on the review of relevant literature, several key aspects related to CBM emerged. It is clear that global and Canadian CBM programs have achieved diverse outcomes, and the expansion of this approach at local and global levels has been remarkable. There is great potential for partnerships since educational institutions participate in CBM projects with substantial skills and resources such as the University of Saskatchewan (SEEP Program at Northern Canada) and the University of Santa Barbara have established research programs to facilitate CBM projects (equipment and technical supports providers).

Indigenous communities have shown a strong interest in supporting CBM, as shown by their involvement in the Canadian Guardian programs and Indigenous Climate Monitoring projects (Natural Resource Canada, 2021). The Government of Canada appears equally committed as evidenced by their $100 million commitment (2021–2026) to support new and existing Indigenous Guardian initiatives (ECCC, 2021). Canadian Indigenous Guardian initiatives attempt to maintain constitutionally protected rights and interests of Indigenous communities, ensuring their empowerment through self-monitoring of their lands and resources (Reed et al., 2020; National Indigenous Fisheries Institute, 2019).

A new report shows that Indigenous Guardian programs have brought positive changes for Indigenous land and peoples (Indigenous Leadership Initiative, 2020). For example, in the Great Slave Lake, the Ni Hat’ni Dene Guardians test water quality in wetlands where tens of thousands of migratory birds raise their young, and the Anishinabek Traditional Ecological Guardians monitor species at risk and climate change impacts within a chain of islands that forms a natural corridor for animals (Indigenous Leadership Initiative, 2020). Regionally, CBM has led to the establishment of informal Indigenous-led advisory groups in Alberta (IWAP, nd) which guide the government of Alberta in respectfully applying traditional ecological knowledge and Indigenous wisdom to Alberta’s Environmental Science Program. Similarly, Canadian Water Rangers programs support local communities’ rapidly expanding participation in aquatic health monitoring by providing training and cost-effective test kits among local communities interested in monitoring their waterbodies (https://waterrangers.ca/). Multi-level cooperation among state departments, NGOs, and northern communities in Canada is expanding. First Nations–led organizations, such as CanNorth (2017) and the Centre for Indigenous Environmental Resources (CIER, 2017), have been working locally and internationally to support CBM projects, suggesting that there are extensive empowerment options through CBM to support local services that are important to address ecosystem problems. Such non-governmental organizations are able to connect local communities with state programs in CBM projects such as northern wildlife monitoring (Anderson et al., 2020; CIER, 2017; CanNorth, 2017). The SWEEP program in northern Canada works as a partnership with the University of Saskatchewan to co-create environmental indicators for fish and aquatic ecosystem health. The use of infographics to identify the ways of monitoring and climate change effects in northern Ontario also advances CBM by demonstrating the value of engaging local communities in ecosystem monitoring assisted by modern drawing tools (Raygorodetsky & Chetkiewicz, 2017, p. 2).

In addition to Canadian cases, there are international projects that track the effects of climate change including monitoring fish, birds, and sea ice in the Arctic Regions (Danielsen et al., 2014). The Rangers program in Australia supports local communities by providing training and jobs (Edwards, 2015; Traill, 2017). The RED + programs involving community-based forest biomass monitoring in developing countries such as Papua New Guinea, Cambodia, Indonesia, Laos, and Vietnam (IGES, 2014) use modern technology like drones and cellphones to train local people to monitor forest biomass (Pocock et al., 2014). Communities in Fiji have raised funds to continue the conservation initiative through their own arrangement and seek self-governance for management of their coastal resources. The creation of virtual maps of the integration of community efforts in Arctic weather, wildlife, and ecosystem monitoring using traditional ecological knowledge has additional significance for the CBM process, as it connects communities (especially Inuit) with international scientists to address ecosystem concerns (Atlas of Community-Based Monitoring & Indigenous Knowledge in a Changing Arctic, 2021; Christie et al., 2008).

This abundance of programs in developed and developing countries covering multiple resource sectors and the continuity of the programs justifies the existence, sustenance, and significance of the CBM approach. The associated advantages of these global and local Canadian programs include social, economic, and ecological outcomes that contribute to sustainability principles as both ecological, social, and economic aspects are considered in CBM projects (Table 3). The social outcomes identified in international cases are communities learning about science (Singh et al., 2014; Trumbull et al., 2000) and forest biomass monitoring (IGES, 2014). The District Toshao Council, Guyana Forest program (Forest Peoples Program Guyana, 2012) empowers communities to control their own forests, and Hawaii’s marine protected areas are being conserved due to CBM. For the Canadian cases, social outcomes are stated as the training offered on standardized water monitoring techniques (Weston & Conrad, 2015). Supporting Indigenous self-governance is another positive social outcome, such as in the case of Canadian Indigenous Guardian Programs (Natcher & Brunet, 2020; Reed et al., 2020; National Indigenous Fisheries Institute, 2019; Wohburg, 2015). The social outcomes of CBM also include providing inexpensive monitoring, communities voluntarily participating in water quality monitoring (Conrad, 2006; Conrad & Hilchey, 2011; Galbarith et al., 2016), and supplying cost-effective data on water quality in Alberta (Alberta Lake Management Society, 2021).

Along with CBM’s successes, this review identified several challenges that affect both global and local cases that need further discussion especially in the context of Canada. Concerns have emerged regarding the attitudes of the scientific community toward the application of the data (Leach, 2018). In the Canadian contexts, unclear authority over the use of CBM data and access arrangements that allow community members to use the information collected through CBM programs present a challenge (Table 4). For example, the absence of an appropriate framework for data sharing and poorly defined intellectual property rights have been sources of dissatisfaction for many communities in northern Saskatchewan (author’s personal experience). Furthermore, negligence in the wider application of data and information gathered through CBM in decision-making stems from the assumption that information collected through this process does not meet scientific standards, which further limits the scope of the process.

Although CBM faces many barriers, they have not stopped the expansion and application of the approach (Fig. 1). For many reasons, CBM can be an effective approach to addressing our pressing ecosystem problems. There are many associated social benefits, such as creation of local jobs, building capacity within communities in data gathering and promotion of local empowerment, which justify the application of CBM. This review also found that the issues and challenges with CBM are neither technical (projects utilize appropriate tools and scientific procedures such as the use of cell-based apps to gather data and transfer the data remotely) nor social (communities want to know about ecosystem health, offer voluntary support and want to participate in science research). Rather, procedural problems hamper the success of CBM, such as the limited use of data by scientific communities in ecosystem modeling, and the insufficient funding and technical commitments of state agencies to local communities. Claims from scientists that question the quality of the data gathered by local users have limited value as poor quality data can be removed from prospective data sets. In addition, science does not provide a complete view of some ecosystem issues, such as the quality of water in a lake affected by eutrophication (Scott, 2015).

Although discussions on the geopolitical environment that affects CBM-driven ecosystem decisions are relevant, there has not been as much focus on this component of ecosystem research so far. In this regard, the issue of Indigenous rights in relation to ecosystem monitoring is relevant. Government decisions must be questioned when program funding becomes intermittent, which is not beneficial for long-term progress of Indigenous communities (Higgins, 2016). For example, assistance for local monitors to succeed and work for their own communities by providing funding through Indigenous Guardians is still uncertain (Reed et al., 2020). Funding application reviewers are seldom members of Indigenous communities and are unable to reflect the needs to be accommodated through CBM projects. For example, Indigenous communities often compete for a small amounts of funds in ecosystem monitoring (Canadian Guardians or Indigenous Community-based Climate Monitoring programs) while aerial surveys with little participation of Indigenous nations is still a favored approach although operation costs of it is fairly high that ranges from $400 to $700/h as per 2003 record (Quellet, 2003) which could be much higher now. Communities are not able to scrutinize the quality of the works done by scientists, and they are not empowered to do so through education and training. Also, they do not participate in scientific analysis of the data generated by such research and therefore, they are unable to contribute to its interpretation. The continued failure to address these issues prevents Indigenous communities from reaping the full benefits of CBM.

Scholars have offered valuable recommendations to overcome the challenges that so often limit the scope of CBM projects and their benefits to Indigenous communities. They have suggested creating measurable monitoring goals, research questions, well-written study designs, clear documentation instructions, and an adequate definition of the scope and complexity of the project to improve the data collection process and its scientific applicability (Green et al., 2016; Conrad, 2006). There is also the question of who actually decides what are considered successes and failures of the CBM process. A simple but limited answer to this is the scientist, as we found little research that examine the satisfaction of the communities involved. From this, it is clear that evaluation of CBM projects is biased toward science. Scientists/managers often evaluate the CBM project outcomes based on criteria that meet their project needs such as data quality or low cost low approach, and not necessarily consider the needs of the community. Promoting CBM by non-Indigenous institutions including government departments to achieve their own goals is problematic.

There are many ways to address the existing concerns that limit CBM throughout the world. One of the most critical steps should involve understanding the communities’ motivations to participate in the CBM process (Pollack & Whitelaw, 2005; Whitelaw et al., 2003). It is beneficial to support collaboration by providing resources (questionnaires, research proposals, etc.) in easy-to-understand formats that utilize local languages and share hard copies of information to overcome a potential lack of Internet access. In this regard, Conrad (2006) suggests improving communication among researchers, communities, and the public to gain support for the CBM process.

Prospective researchers must also consider the impact of the tools/methods they seek to use on Indigenous communities. Authors have recommended using technology such as drone-based monitoring, smartphone-based apps, and photo-voice techniques for real-time data collection and tool kits as an effective way to limit the cost of monitoring, increase efficiency, and ensure accurate collection of high-quality data in CBM projects (IGES, 2014; Johnson et al., 2016; Andrachuk & Armitage, 2015). However, use of such technologies may reduce jobs for Indigenous people in already poverty-prone areas with few employment opportunities, such as remote Northern communities. Offering community incentives such as ownership of equipment (trail cameras, multi-meters, or similar monitoring devices) and research benefit sharing arrangements may ensure Indigenous participation while enabling the use of technology. Enlisting the help of the community to produce detailed visualizations of Indigenous knowledge-based monitoring CBM outcomes through infographic technologies (Raygorodetsky & Chetkiewicz, 2017) can also engender community interest to take part in science research (Wildlife Society of Canada’s infographic exercise, 2018).

In addition, poorly defined protocols for ethical use of data, ownership, and intellectual property rights are recognized as an obstacle to CBM especially in relation to Traditional knowledge application (Scassa & Taylor, 2017). Ensuring ownership of the CBM data to avoid its commoditization can minimize these concerns. More progressive recommendations involve maintaining networks of participants. For example, Sharpe and Conrad (2006) support building monitoring networks to share knowledge about CBM projects, which would consist of local and regional groups that encourage dialogue and collaboration among communities and scientists. To increase the accessibility of information from data collection organizations such as aquatic program management departments, scholars propose establishing local and regional data hubs sourced from Indigenous knowledge, which can invite CBM managers, industry, governments, and research institutions as guests (see Kanu et al., 2016, p. 6). Creating community-controlled central databases or archival systems can provide ownership of the data by the source communities.

Canadian CBM issues should be addressed separately, given the colonial aspects of resource governance with respect to the rights of Indigenous communities over their traditional lands and their visibility in CBM (Johnson et al., 2016; Reed et al., 2020). In the Canadian context, the influences of colonial powers on CBM should be removed by recognizing the autonomy of the knowledge holders in making decisions about their land and resource management projects (Carlson et al., 2017; Reed et al., 2020). As per Eicken et al. (2021), respecting the rights of participating Indigenous and local communities should be a central aspect of all CBM programs and is critical to successful co-design and co-creation between top-down (state managed) and bottom-up (community focused) approaches. Moreover, researchers in Canada can increase community interest by creating job opportunities for Indigenous people by promoting land-based learning and engaging members in long-term monitoring as is done in the Australian Rangers programs (Australian Government, 2017; Peters et al., 2016).

A recent research study forecasts the strong potential of CBM as a tool for sustainable Indigenous self-determination (Reed et al., 2020). In this regard, scientists should not view CBM solely as a low-cost approach to collecting and sharing data and traditional knowledge, as it is very disrespectful to the Indigenous communities. The data used by scientists are gathered from the extensive history of the community with the cooperation of local Indigenous members. There should be a standard approach to supporting the CBM process through adequate remunerations and acknowledgment of the cultural heritage of Indigenous Nations exemplified by this knowledge. This need for fair CBM program compensation is applicable for all communities across the globe.

In order for Canadian CBM programs to function effectively, scientists must also consider the potential long-term benefits to the Indigenous community in the form of youth training in data collection, analysis and reporting, and science education. Implementation of these approaches may address both the scientific and social shortcomings of CBM as discussed, while enhancing its utility as an effective method of ecosystem monitoring. Scientists working in CBM can also consider the provisions created under the Canadian Impact Assessment Act (2019) for the use of Indigenous knowledge as a guide to managing Indigenous lands in CBM projects, and more clearly, they can use CBM approaches to support for Indigenous sustainable self-determination (Reed et al., 2020).

As per our knowledge, CBM is not welcomed by many Indigenous communities in Canada, especially when it is treated solely as a research project and not as a process to support the Indigenous community on a long-term basis. A general expectation from the Indigenous Nations participating in CBM is that their youth are trained in science research methods. The absence of youth-targeted objectives in CBM projects can be considered a missed opportunity to support the reconciliation process by enhancing science education in these communities (Schaefer, 2012). As per present information, Indigenous youth has the lowest participation in science education in comparison to the non-Indigenous youth (Wong et al., 2020).

A recent study by Wong et al. (2020) discusses ten provisions (or calls to action) to overcome the challenges facing Indigenous youth science education in Canada. These provisions can be readily adapted to address the challenges that we have identified in the CBM process, namely, increasing community involvement at all stages and respecting Indigenous autonomy. Wong et al. recognize the importance of understanding existing socio-political contexts and creating a space for effective collaboration and knowledge co-creation when implementing science programs. They recommend that such programs provide opportunities for youth who are trained in both TEK and natural science, and be connected to cultural revitalization. They also recommend Indigenous involvement in the program funding review process and in the selection of programs they consider appropriate for their communities. Wong et al. further emphasize the need for proper acknowledgement of Indigenous rights over their knowledge from researchers and academic journals that intend to publish manuscripts utilizing traditional knowledge systems. Together, these provisions provide a framework for implementing CBM projects in a manner that minimizes negative impacts while providing maximum benefits to the communities. If carried out with these considerations, CBM can be used as a means to empower Indigenous people.

Evaluating the local and global cases, it is understandable that although CBM approach is facing certain obstacles, it is somewhat the last resort to address environmental crises that are on the rise with the global population increase and also due to the climatic change such as wildfires that contribute to the ecosystem disturbance. Community empowerment through educating them with a focus on Indigenous Nations in environmental data-gathering process aided by modern technologies (e.g., use of cellphones, drones etc.), support data logging using online platforms and help them in data sharing (certainly by maintaining ethical aspects and Indigenous protocols) among the CBM project participating communities and beyond, and finally resolving the funding issues will be the keys to achieve the growing needs of CBM.

## Supplementary Information

Below is the link to the electronic supplementary material.

Supplementary file1 (DOCX 8 KB)

## Data Availability

We used open access data available through desktop search for developing the manuscript. For additional information regarding Indigenous community–based monitoring programs, see the links included in the Supplementary Index below.
