# Supplementary material for: The promise and pitfalls of community-based monitoring with a focus on Canadian examples
Source: Environ Monit Assess. 2023 Mar 6;195(4):445. doi: 10.1007/s10661-022-10841-y (PMC9988817; doi:10.1007/s10661-022-10841-y)
Supplement: Supplementary file 1 — Supplementary file1 (DOCX 8 KB) [file 10661_2022_10841_MOESM1_ESM.docx]

**Supplementary Index:**

1. Canadian Indgenous Gaurdian Programs: https://www.indigenousguardianstoolkit.ca/program-map
2. Community-based monitoring in the NWT (Northwest Territories): Working with 21 communities to monitor water quality at over 40 sites on 24 NWT rivers and lakes. <https://www.enr.gov.nt.ca/en/services/water-management-and-monitoring/community-based-monitoring>
3. Slave River and Delta Partnership- NWT Water Stewardship

<https://www.nwtwaterstewardship.ca/sites/water/files/resources/128-cbm_water_quality_results-press.pdf> and <https://www.researchgate.net/figure/Map-of-the-Peace-Athabasca-Delta-showing-locations-of-the-lake-circles-and-river_fig1_343872259>

1. Lake Watch Alberta’s Water quality monitoring sites: <https://www.alberta.ca/assets/images/afred-lake-trophic-indicator-figure-2.jpg>
2. Center for Indigenous Envirmental Resources community-based monitoring project locations: https://yourcier.org/what-we-do/
3. USA Volunteer Water Monitoring Network : <http://www.volunteermonitoring.org/programs>
4. Fish Forever global locations: <https://rare.org/program/fish-forever/>
5. Australian Indigenous rangers programs: <https://theconversation.com/indigenous-ranger-programs-are-working-in-queensland-they-should-be-expanded-89766>; <https://www.clc.org.au/our-rangers/>
6. UN REDD+ projects in Africa, the Asia-Pacific, the Caribbean and Latin-America: https://www.surinameredd.org/en/reddplus/countries/
